# Can Continuous Levodopa Delivery Be Achieved in the Absence of Intrajejunal Levodopa Infusion? Implications for India and Underserved Countries

**DOI:** 10.1002/mdc3.13915

**Published:** 2023-11-28

**Authors:** K. Ray Chaudhuri, Lucia Batzu

**Affiliations:** ^1^ Department of Basic and Clinical Neuroscience Institute of Psychiatry, Psychology, and Neuroscience, King's College London London UK; ^2^ Parkinson's Foundation Centre of Excellence, King's College Hospital London UK

**Keywords:** continuous dopaminergic stimulation, low and middle income countries, Parkinson's, underrepresented populations

Continuous drug delivery (CDD) in Parkinson's disease (PD) aims to achieve continuous dopaminergic stimulation (CDS) and, in theory, aims to mimic physiological tonic, rather than phasic, stimulation of the striatal dopamine receptors.^1^ In animal models, the concept has been shown to substantially reduce motor fluctuations and specifically dyskinesias; although in humans the evidence has been less robust, as dyskinesias or “off” periods break through in the long term.^2^ Physiologically, the tonic firing of dopaminergic neurons in the substantia nigra pars compacta ensures that striatal dopamine levels are maintained relatively constant.^3^, ^4^ However, the pulsatile phasic dopaminergic stimulation given by intermittent oral levodopa intake induces molecular and physiological changes in the neurons of the basal ganglia, and this is considered primarily responsible for the development of motor complications such as dyskinesia and “wearing off”.^5^, ^6^

Oral levodopa remains the mainstay for treatment of PD globally, although estimates from 2019 showed over 8.5 million individuals living with PD worldwide, prevalence that had doubled over the previous 25 years.^7^, ^8^ The number of people with PD is expected to reach over 12 million by 2040,^9^ and the increase will affect mainly developing countries, with escalating costs of care. PD is an age‐related, progressive disorder, and progression to an advanced stage is inevitable. The cost of advanced PD, which ideally requires bespoke advanced therapies, as discussed below, is therefore, likely to be massive (~$40,800 USD per patient/year) as based on Western calculations^10^ (Fig. 1). This cost would be unaffordable in most developing regions such as Africa, India and Southern Asia, China, and South America, where the maximum impact of the growing number of patients with PD would be mostly felt. Alternative options or cheaper therapies are, therefore, increasingly important to initiate to address this burgeoning inequality in healthcare.

**FIG. 1:**
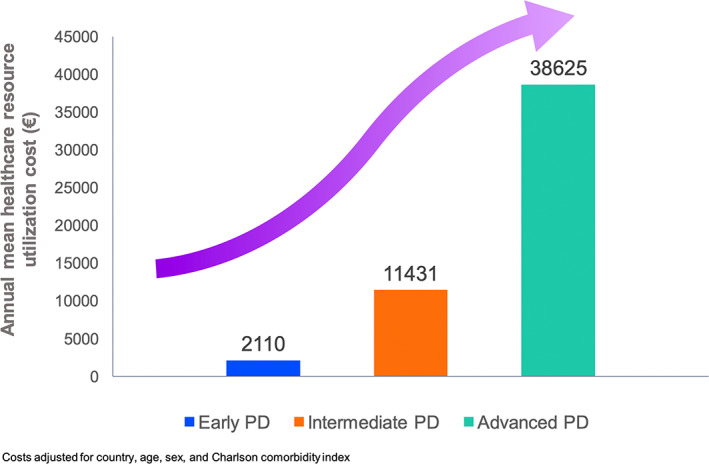
Annual healthcare costs are significantly higher for patients with advanced PD in EU5 countries (Spain, Italy, United Kingdom, France, and Germany).^10^ PD, Parkinson's disease.

## Levodopa‐Based and Other CDD Strategies

Following the use of optimization of levodopa therapy through enzyme inhibition and long‐acting dopamine agonists to address the problems of pulsatile therapies in PD, several device‐aided therapy options have been developed to provide, at least in part, CDD to patients with motor complications. The mechanisms appear to be linked to the concept of bypassing the gastrointestinal (GI) barriers to oral absorption and transport of levodopa.^11^ Subcutaneous apomorphine infusion,^12^, ^13^ for example, has been classified in the United Kingdom (UK) as medical therapy able to provide CDS to tackle fluctuations and dyskinesias.

Another highly effective device‐aided CDD option is the minor surgery‐enabled levodopa‐carbidopa intestinal gel (LCIG) infusion,^14^ together with the more recent levodopa‐entacapone‐carbidopa intestinal gel (LECIGON) infusion, which is a combination of levodopa, carbidopa monohydrate, and the catechol‐*O*‐methyltransferase (COMT) inhibitor entacapone.^15^, ^16^ Deep brain stimulation (DBS) of the subthalamic nucleus or the pars interna of the globus pallidus^17^ is another invasive option that has been in use since the 1990s and is available in most countries only to the affluent patients who can afford the cost of this treatment.

Despite being developed in the 1960s, levodopa remains the most effective therapy for PD. It is affordable to a global PD population and not only to citizens of developed and wealthy countries, as applicable to the current device‐aided therapies. A discussion on latest developments in levodopa delivery and its affordability is, therefore, relevant in a global context. LCIG infusion, in particular, is one of the most effective therapies in advanced PD, with recent data suggesting efficacy shown for at least 13 years in advanced PD as well as in a small cohort of parkinsonian patients with progressive supranuclear palsy.^18^, ^19^ Next to these established treatment strategies, several new device‐aided therapies have been tested in phase 3 randomized controlled trials with positive results and wait formal licensing. These include levodopa‐entacapone‐carbidopa intestinal gel (LECIG) infusion (already approved in several countries), subcutaneous levodopa/carbidopa,^20^ and subcutaneous foslevodopa/foscarbidopa preparations,^20^, ^21^, ^22^ which are soon to enter the market with the aim to mimic CDS.

Non‐levodopa formulations that may be able to provide CDD and in current use include apomorphine subcutaneous infusion and transdermal rotigotine patch, but they will not be discussed in detail in this article.

## The Economic Burden of CDD

The progress made in CDD with device‐aided therapies comes at high costs and requires healthcare systems able to absorb the highly expensive maintenance of such therapies, making these available only in selected, high‐income countries, and to affluent patients of underserved countries. If one considers the patient community in India as an exemplar for a lower‐middle‐income country, the cost for a 500 mg/day regimen of oral levodopa is ~$60 USD/year. However, the average salary in India ranges approximately from $1660 to $2680 USD/year,^23^ with a minimum national wage of approximately $760 USD/year.^24^ Unless government funded, even oral levodopa therapy may be unaffordable to many Indian patients. Therefore, device aided therapies remain out of bounds for most such patients, although clinical needs are inevitable. As an example, the approximate cost of apomorphine therapy in India ranges between $3665 and $4887 USD/year, whereas, for DBS, the overall expenditure for a DBS procedure with a rechargeable device and maintenance with an average of 15 years of life is ~$1833 to $2444 USD/year (information gathered by local experts). Moreover, the cost of LCIG infusion per patient per year in India is equivalent to $39,075 USD/year,^25^ hence, potentially prohibitive, despite being considered the most effective form of levodopa replacement in advanced PD. In addition, on average, 300 new patients attend a movement disorder clinic every year^26^ in India and, as such, the economic burden of advanced therapies makes these unaffordable for most patients. In essence, the vast majority of advanced patients with PD in underserved countries such as India remains, and continues to remain, inadequately treated. This fact shines a light on the growing issue of inequalities in the access to optimal and bespoke healthcare, which are driven by poverty, scarcity of resources, and by the soaring costs charged by pharmacological industry for their products and technology.

## Existing Inequalities In Research and Healthcare

**FIG. 2:**
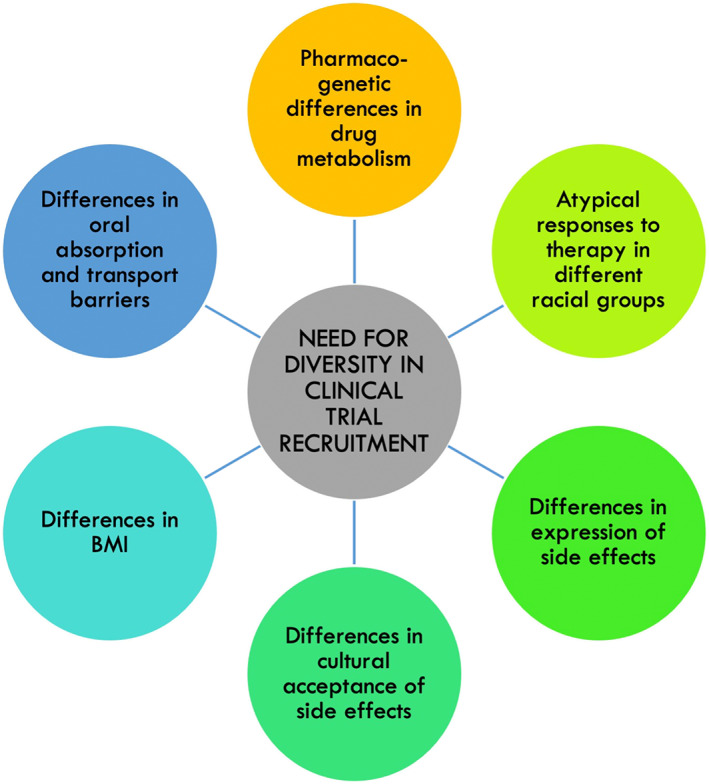
The need for diversity in Parkinson's disease clinical research and its determinants. BMI, body mass index.

Another aspect to consider while addressing inequalities of care in the context of CDD is that even the most recent long‐acting levodopa‐based treatments, such as IPX‐203 (a new extended release levodopa/carbidopa),^27^ subcutaneous levodopa/carbidopa or foslevodopa/foscarbidopa,^28^ as well as the Accordion Pill (delivery based on gastric retention of multilayer films containing immediate‐ and controlled‐release levodopa/carbidopa)^29^ among others, have been investigated in clinical trials recruiting almost entirely White patients, with a small number of patients belonging to ethnic minorities.^30^ This has been recently highlighted by a study conducted by Lau and colleagues,^30^ showing that the inclusion of under‐represented ethnic minorities groups in recently published clinical trials for PD is only 21.57%, and is not even considered in most studies. This constitutes a severe underrepresentation when compared to the proportion, for example, of Black or African American in the UK and United States (US) population.^30^ Other examples include clinical trials addressing cognitive impairment in PD conducted in the past 5 years, where more than 90% of recruited participants were White and non‐Hispanic.^31^ These findings add to the already existent inequalities in research in PD where, for example, also women are underrepresented beyond what expected from the lower prevalence of PD in this group.^32^ Ethnic disparities of care are also observed in daily clinical practice, as demonstrated by the reduced prescription of analgesics for PD‐related pain in Black patients when compared to White patients with similar pain ratings.^33^ Apart from the societal relevance of inequality, there may also be pharmacogenetic, cultural, and other racial differences in drug metabolism and eligibility for treatment (as highlighted during the coronavirus disease 2019 pandemic)^34^ and these cannot be addressed by clinical trials conducted in the White population only (Fig. 2).

## A Four‐Level Solution

**FIG. 3:**
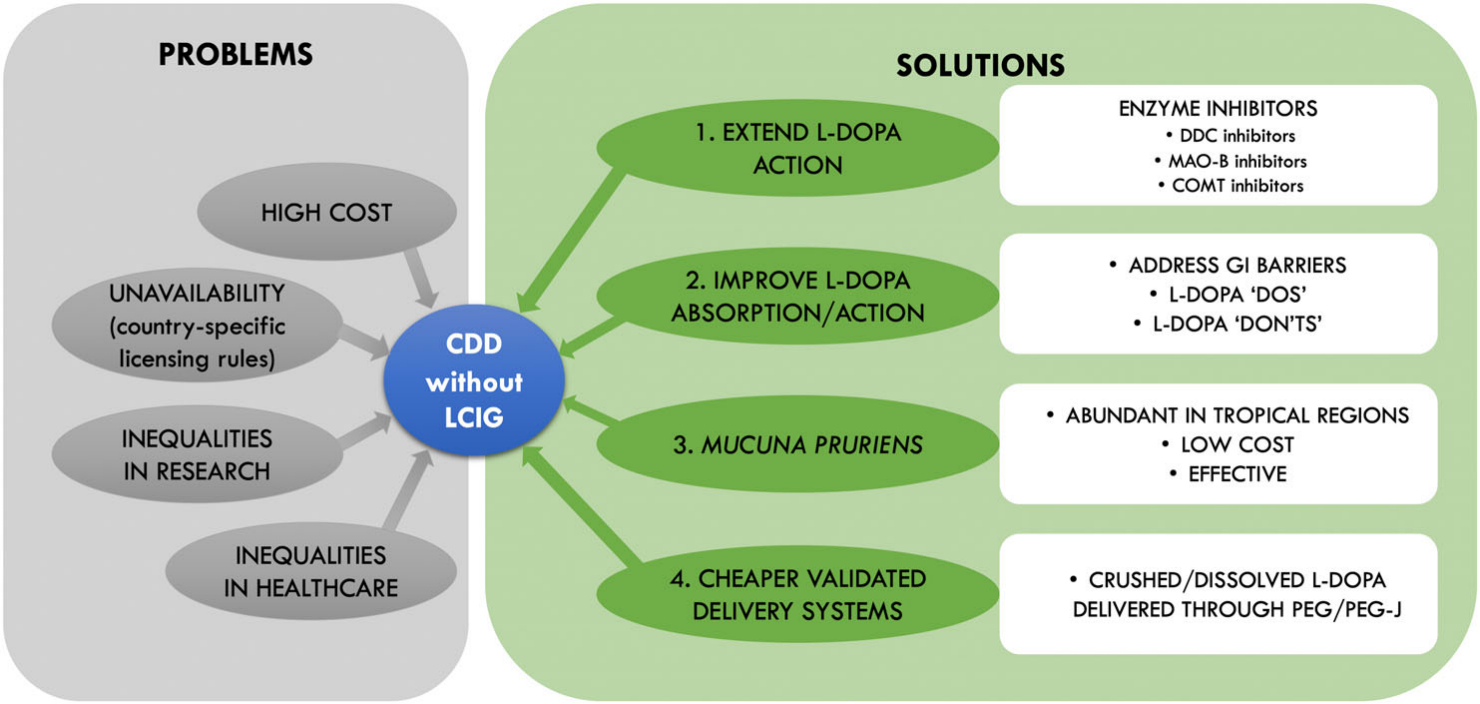
Problems related to continuous drug delivery in the absence of levodopa infusion therapies and possible solutions. CDD, continuous drug delivery; LCIG, levodopa/carbidopa intestinal gel; L‐DOPA, levodopa; DDC, dopa decarboxylase; MAO‐B, monoamine oxidase type B; COMT, catechol‐*O*‐methyltransferase; GI, gastrointestinal; PEG, percutaneous endoscopic transgastric gastrostomy; PEG‐J, percutaneous endoscopic transgastric jejunostomy.

How can, therefore, the massive and glaring inequalities of care in the context of levodopa‐based CDD strategies denying bespoke advanced therapies to hundreds of patients in need be addressed? One potential strategy may be to explore whether levodopa‐based CDS can be achieved in the absence of expensive therapies. We propose a four‐level solution encompassing the following: (1) extend the action of levodopa; (2) improve the absorption and transport of levodopa; (3) consider the use of *Mucuna pruriens* (MP); and (4) reduce the cost of validated delivery systems (Fig. 3 **).**

### Level 1: Extend Levodopa Action

Levodopa and dopamine are metabolized by several enzymes, such as dopa decarboxylase (DDC), COMT, monoamine oxidase (MAO), and aldehyde dehydrogenase (ALDH), causing overall peripheral and central degradation and reduction of dopamine available to the nigrostriatal pathway.^35^ Carbidopa and benserazide were developed to counteract the action of the peripheral DDC responsible for the conversion of levodopa to dopamine at the systemic level.^36^ DDC inhibitors are an integral part of any levodopa‐based regimen, being responsible for a 10‐fold increase in central nervous system levodopa availability.^35^ Oral levodopa with DDC inhibitors is available globally and is often the only therapy available to PD patients. Central MAO type B and COMT enzyme inhibitors are also in widespread use in developed countries. Among MAO‐B inhibitors, rasagiline is the most available and commonly used because of lack of amphetamine‐like activity or affinity for any of the catecholaminergic or serotonergic receptor groups, compared to selegiline.^35^ In addition, a controversial preclinical and clinical evidence suggest a potential neuroprotective role for rasagiline.^37^, ^38^ COMT inhibitors have also been used to increase the “on” time while managing motor fluctuations in patients with PD. In particular, the second‐generation inhibitor entacapone and, more recently, the third‐generation opicapone have become the most used because of their effectiveness and lack of serious adverse events such as liver toxicity. Opicapone, in addition, has increased power, longer action, and better pharmacokinetic and safety profile compared to previous COMT inhibitors.^39^, ^40^, ^41^ However, it is expensive and unlikely to be affordable for patients in developing countries. Improving the overall bioavailability of levodopa with the appropriate use of MAO‐B and COMT inhibitors has, therefore, the ability to provide a dopaminergic stimulation profile that is more stable and semi‐continuous, therefore, mimicking, at least in part, the CDD stimulation modality. In underserved areas, however, only compounds that are now available in generic forms may be available for prescription. In India, rasagiline, selegiline, safinamide, and entacapone are currently available. Local and learned society‐driven clinical guidelines should provide guidance on this strategy.

### Level 2: Improve Levodopa Absorption and Transport

Levodopa is characterized by limited oral bioavailability and extremely short half‐life, therefore, representing one of the most difficult molecules to control pharmacologically.^11^ Part of its limited bioavailability, as previously discussed, lies in the numerous metabolic pathways and enzymatic degradations encountered across its journey from the GI tract to the brain, beyond the blood–brain barrier. GI dysfunction, in addition, is a cardinal non‐motor symptom^42^ and a feature of the recently described noradrenergic subtype of PD,^43^ which adds to the overall challenge of levodopa absorption when administered orally, due to both absorption as well as transport barriers. Addressing these obstacles with relatively cheap lifestyle‐ and diet‐based advice would, therefore, result in better bioavailability of oral levodopa and, subsequently, in a less discontinuous dopaminergic stimulation.^11^ Barriers to absorption are represented by the action of human and bacterial decarboxylases,^44^, ^45^ the saturable facilitated transport system, where levodopa competes with other dietary amino acids,^46^ the peripheral COMT action, and small intestinal bacterial overgrowth.^47^ In addition, *Helicobacter pylori* (*H. pylori*) infection may impair levodopa absorption by various mechanisms, although the role of its eradication in improving the motor effect of oral levodopa is disputed, there being evidence both for and against.^11^, ^48^ GI barriers to levodopa transport include dysphagia, esophageal alterations, delayed gastric emptying,^49^ slow transit, and outlet constipation.^11^ Prompt management of these issues, together with *H. pylori* screening and eradication, can therefore, prove to be an effective strategy to improve the bioavailability of levodopa.

**FIG. 4:**
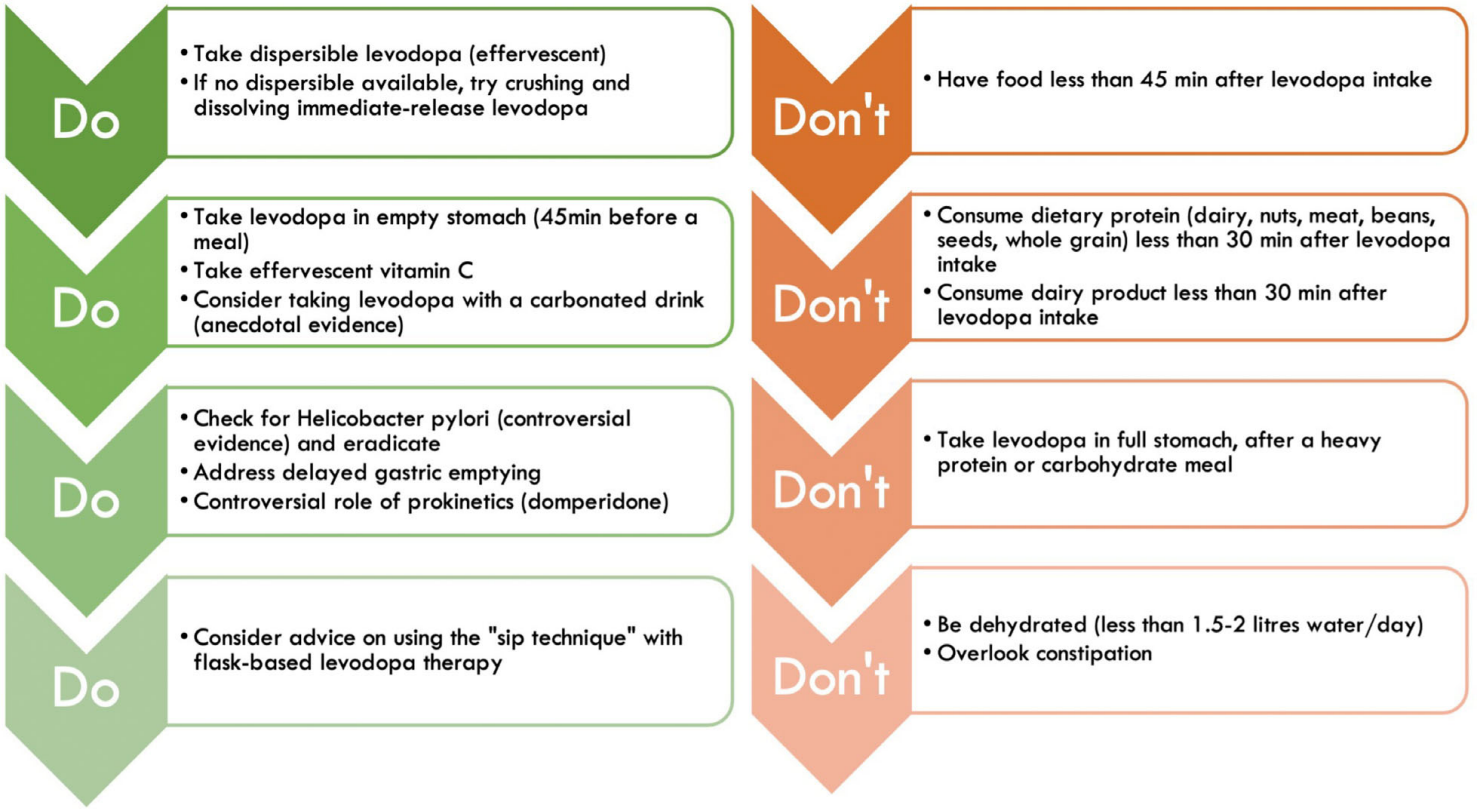
“Dos” and “don'ts” of levodopa therapy.

**FIG. 5:**
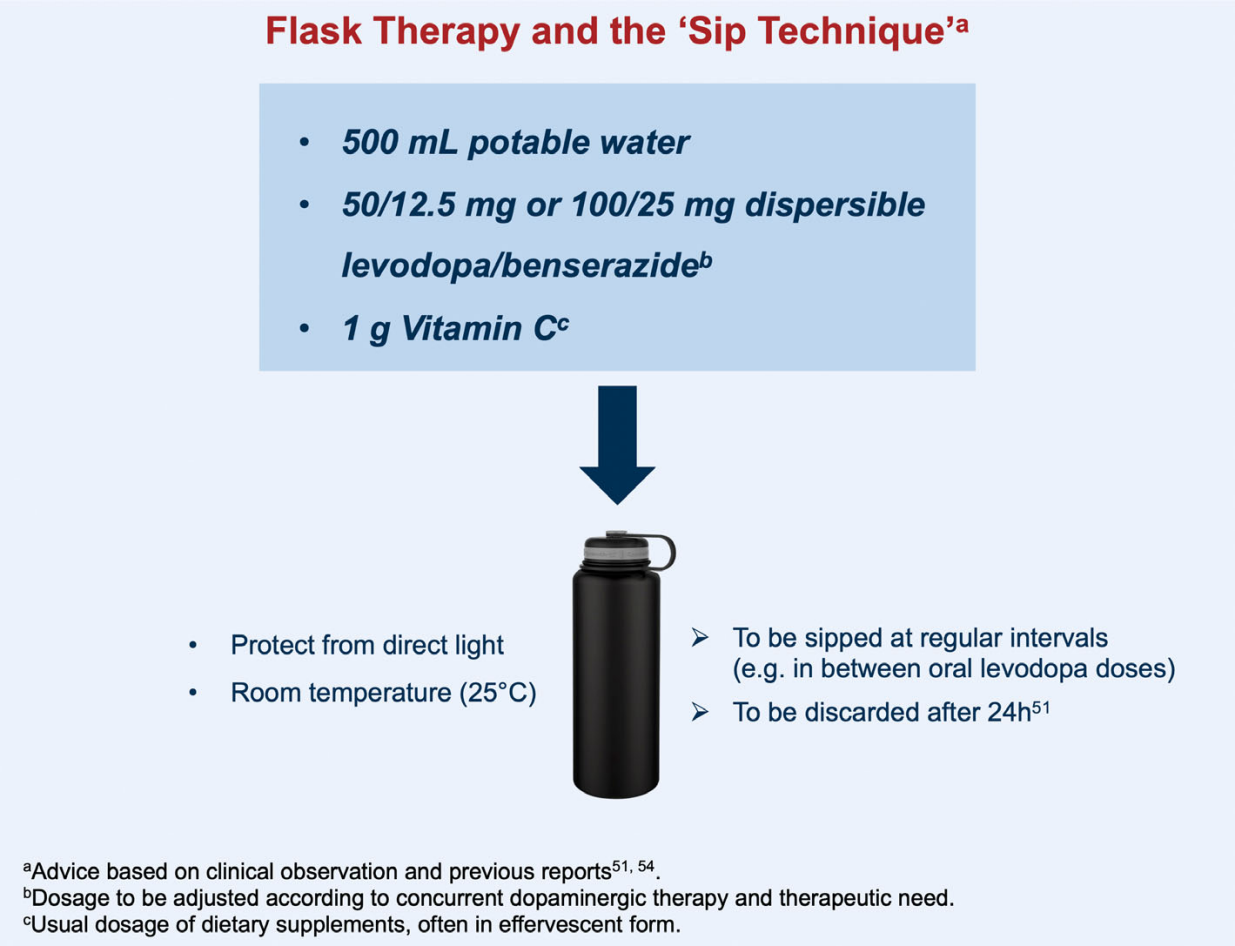
The potential use of the flask‐based therapy and “sip technique”.

Further contribution to this issue can be given by the identification of “dos and don'ts” in relation to levodopa therapy and food, therefore, triggering specific dietary and lifestyle interventions (Fig. 4). Among the “dos”, for example, the intake of levodopa, particularly in the dispersible form, with orange juice or carbonated drinks augmented by dispersible vitamin C tablets,^50^ may facilitate its overall absorption. Another possible “do” is represented by the “sip technique” and flask‐based therapy, consisting in the intake of dispersible levodopa/carbidopa dissolved in water,^51^ stored in a flask protected from direct light and at room temperature, and sipped at regular intervals and/or at night (Fig. 5). This may address the “delayed on” or the “no on” periods, as well as nocturnal akinesia (Chaudhuri and Batzu, personal observation). This practical advice is based on the evidence that the dispersible formulation of levodopa has shorter time to peak plasma level compared to the standard formulation.^15^, ^52^, ^53^, ^54^ It is important to note, however, that this technique has some limitations, including reduced stability with high temperatures and variability of levodopa dose associated with each “sip.” Relevant “don'ts” in the levodopa‐food relationship include food consumption, and particularly proteins, less than 30 minutes before or after levodopa intake,^11^ meals with high protein content,^55^ and diet with high dairy intake.^56^

### Level 3: *Mucuna Pruriens*

Despite being the primary treatment for PD for over 50 years, the global standard of levodopa with a DCC inhibitor remains unavailable and/or unaffordable in many Sub‐Saharan African countries and several other low‐income countries.^57^, ^58^ MP is a wild leguminous plant commonly found in tropical regions, containing a levodopa concentration between 0.81% and 9.49%.^59^, ^60^ It is commonly found at low cost in local markets as soil fertilizer or as a remedy against male infertility.^61^ It has recently been proposed the use of MP to replace, or as add‐on to, other dopaminergic treatments because of its low cost and wide availabilty.^61^ Because of its recent re‐discovery in the context of natural remedies and non‐conventional therapies for PD, MP is also often sold in high income countries at high costs. In recent years, however, its potential as affordable and easy‐to‐produce treatment for PD where levodopa is unavailable has initiated several research studies aiming to prove the feasibility, efficacy, and cost effectiveness of MP‐based therapeutic regimens. It has been estimated that 7 g of MP roasted powder obtained without any pharmacological processing is able to provide 400 mg of DDC inhibitor‐free levodopa, approximately equivalent to 100/25 mg of levodopa/carbidopa, for a total annual cost per patient of $10 to $15 USD (against ~$365 USD/year for standard levodopa/DDC inhibitor in Sub‐Saharan Africa and a population living with less than $2 USD/day).^59^, ^62^ In a double‐blind, randomized, controlled crossover trial, single‐dose MP intake met all non‐inferiority efficacy and safety outcome measures in comparison to dispersible levodopa/benserazide.^63^ In addition, high‐dose MP (levodopa concentration of 17.5 mg/kg) gave similar clinical effects to the same dose of levodopa alone, with better tolerability profile.^63^

MP, however, is also available online and the prescriber needs to be aware of the dose conversions that relate to commercial levodopa.

### Level 4: Making CDD More Affordable

Another option to consider when addressing the need of CDD in the absence of device‐aided therapies is the potential delivery of dispersible levodopa/benserazide as liquid solution by a syringe through a percutaneous endoscopic transgastric gastrostomy (PEG) or jejunostomy (PEG‐J). PEG‐Js are currently performed to allow LCIG infusion through an expensive pump system, but the cost of the procedure only, despite being a minor surgery, is not prohibitive. In this way, when liquid levodopa is provided at close time intervals, effects similar to LCIG may be observed, therefore, mimicking CDD. In the context of administering the drug beyond the stomach (as in the case of a PEG‐J), dilution of the medication with sterile water is advised.^64^ Tablets can be dispersed in 10 mL of water to give a cloudy white dispersion and particular attention should be given in avoiding concomitant food intake.^64^

Furthermore, increasing the affordability and accessibility to device‐aided CDD in underserved areas might benefit from the exploration of additional strategies: localized manufacturing, governmental support mechanisms, and integration within insurance frameworks may provide insights into innovative approaches that may alleviate the economic barriers associated with the adoption of device‐aided therapies such as LCIG. For example, when considering India's recognized status as global hub for pharmacological manufacturing, the prospect of local production while maintaining high quality presents a substantial opportunity to significantly reduce associated costs. In such scenario, local community entities, support organizations, and patient groups can also play an instrumental role in advocating for policy reforms that prioritize subsidized pricing or even cost‐free provisions of CDD through government‐affiliated healthcare centers.

## Alternatives To Levodopa

Despite their important role in providing CDD, apomorphine subcutaneous infusion and rotigotine transdermal patch are still expensive in the context of developing countries and mostly not available or, especially in the case of rotigotine, often available through alternative channels. However, there is the potential to develop local technologies for the production of rotigotine and apomorphine, particularly considering the relatively lower manufacturing costs associated with apomorphine and the related infusion system.  Indeed, apomorphine was originally developed for clinical use in the United Kingdom when local pharmacies prepared and delivered apomorphine for clinical studies reported in the 1980s and early 1990s.^65^

Of note, strategies aimed at reducing DBS costs and the utilization of alternative, more affordable surgical therapies can contribute to optimizing the management of patients in need of continuous treatment. For instance, the cost‐effectiveness of non‐rechargeable implantable pulse generators in comparison to rechargeable counterparts may facilitate the dissemination of DBS within low‐income regions.^66^ Moreover, although there is a preference for DBS and newer techniques like magnetic resonance imaging‐guided focused ultrasound in higher‐income nations, other ablative surgical techniques, such as radiofrequency thermoablation, and stereotactic radiosurgery, should be considered in situations where patients need to self‐finance, as often occurs in lower‐middle‐income countries.

## Conclusions

Reducing inequalities in healthcare should be a primary objective for any healthcare policy, learned society, and patient charity. PD is the fastest growing neurodegenerative disorder and the highest increase in prevalence will involve lower‐middle and low‐income countries, where life expectancy has increased in the past decades. In this scenario, it is irresponsible to ignore the urgent need to address not only the basic aspects of PD‐related management, but also the challenges of advanced PD, including motor and non‐motor fluctuations and, therefore, the necessity of making CDD available. Although clinical guidelines based on Western and developed countries espouse the use of advanced therapies based on clinical criteria, the unavailability of such therapies in vast parts of the world and to needy patients is often ignored. The solution to this problem is not an easy one, and may require a financial and political decision‐making process with relevant stakeholders such as industry, policy‐making bodies as well as patient advocacy groups. Although policy makers and healthcare systems adapt to these long‐term objectives, it is essential to provide acceptable and realistic options in the meantime. We have, therefore, proposed a few, essential strategies that can support the need for CDD and, more in general, optimal dopaminergic stimulation in the absence of LCIG and other device‐aided therapies. We suggest researchers to undertake clinical trials using some of these strategies to provide robust evidence for management, as it has been shown in the MP studies. Research should also take the role of tackling the current barriers in trial participation for underserved communities and low‐income areas with, for example, increased funding directed to bespoke local outreach strategies and direct contact with primary care physicians to overcome the barriers of sporadic specialist referrals. Increasingly large industry manufacturers of molecules to manage PD need to be aware of health inequalities issues and address pricing of medications so that costs are not prohibitive to a vast number of PD patients in the world. This issue currently remains a major unmet need, where learned societies and government policy makers can play an important part. In addition, industry stakeholders have also the responsibility to address recruitment inequalities in clinical trials for PD and the lack of data in many underserved countries. In a similar way, academic institutions need to consider investigator‐led clinical trials looking at feasible, real‐life, culturally bespoke, and country‐specific therapies that can benefit thousands of patients worldwide. It is a “long and winding road” and yet a necessary one toward real equality and treatment opportunities for all people with Parkinson's disease.[Fig mdc313915-fig-0006]

**VIDEO 1 mdc313915-fig-0006:** 

## Author Roles

(1) Project: A. Conception, B. Organization, C. Execution; (2) Manuscript Preparation: A. Writing of the first draft, B. Review and Critique.

K.R.C.: 1A, 1B, 1C, 2A, 2B

L.B.: 1B, 1C, 2A, 2B

## Disclosures

**Ethical Compliance Statement:** The authors confirm that the approval of an institutional review board and informed patient consent were not required for this work. We confirm that we have read the Journal's position on issues involved in ethical publication and affirm that this work is consistent with those guidelines.

**Funding Sources and Conflicts of Interest:** No specific funding was received for this work. The authors declare that there are no conflicts of interest relevant to this work.

**Financial Disclosures for the previous 12 months:** K.R.C. declares that he has received honoraria for advisory board membership from AbbVie, UCB, GKC, Bial, Cynapsus, Lobsor, Stada, Zambon, Profile Pharma, Synovion, Roche, Therevance, Scion, Britannia, Acadia, and 4D Pharma; speaker's honoraria for lectures from AbbVie, Britannia, UCB, Zambon, Novartis, Boeringer Ingelheim, Bial, Kyowa Kirin, SK Pharma, Scion, GKC, MDS, and EAN; investigator initiated research grants from Bial; academic grant funding from EU Horizon 2020, Parkinson's UK, National Institute for Health and Care Research (NIHR), Parkinson's Foundation, and Wellcome Trust; royalty payments or license fees from Oxford University Press Cambridge University Press, MAPI institute (King's Parkinson Disease Pain Scale and Parkinson Disease Sleep Scale 2); and payment for expert testimony from the General Medical Council GMC, NICE, and NIHR unrelated to the submitted work. L.B. declares that she has received speaker's honoraria for lectures from AbbVie.
